# Editorial: The IV Latin American Metabolic Profiling Society (LAMPS) symposium: 2022

**DOI:** 10.3389/fmolb.2024.1430789

**Published:** 2024-06-21

**Authors:** Martín Arán, Pablo A. Hoijemberg, Guillermo Moyna

**Affiliations:** ^1^ Laboratorio de RMN, Fundación Instituto Leloir (IIBBA-CONICET), Buenos Aires, Argentina; ^2^ Centro de Investigaciones en Bionanociencias (CIBION-CONICET), Buenos Aires, Argentina; ^3^ Departamento de Química Del Litoral, CENUR Litoral Norte, Universidad de La República, Paysandú, Uruguay

**Keywords:** Latin America, Latin American Metabolic Profiling Society, metabolomics, nuclear magnetic resonance, mass spectrometry

In the past two decades, metabolomic analysis has evolved from being an important tool to becoming a standalone discipline. It has been applied to a wide variety of problems, including clinical diagnosis and biomarker identification, crop selection and protection, and food authentication, among others. Most of the advances in metabolomics have gone hand in hand with the standardization of methods and protocols, as well as with advances in analytical instrumentation and bioinformatics tools capable of handling large amounts of samples and data. The latter are inherently associated with considerable investments in resources, putting researchers in emerging regions such as Latin America at a clear disadvantage. However, the number of research groups, institutions, and dedicated facilities focusing on metabolomic analysis has increased modestly but steadily in the past years in Latin American countries. One of the catalysts of this growth has been the Latin American Metabolic Profiling Society (LAMPS), an organization founded initially by researchers in Argentina, Colombia, Brazil, Peru, and Uruguay in the mid 2010s. Since its early days, LAMPS has promoted collaborative research projects between members and fostered training opportunities for students and young investigators, and has also organized biennial meetings attended by researchers in the field from Latin America and the rest of the World. The first LAMPS meeting was held in Lima, Perú (2014), and gatherings in Rosario, Argentina (2016) and Rio de Janeiro, Brazil (2018) followed. The fourth LAMPS meeting planned for 2020 was postponed due to the COVID-19 pandemic, and was then celebrated in Cartagena de Indias, Colombia, in 2022. With 14 invited panelists and over 150 participants, this proved to be the largest LAMPS gathering so far.

To partly document this continuing growth, this Research Topic collects manuscripts from Latin American groups that participated in the IV LAMPS. As evidenced in the volume, the contributions describe the use of metabolomics analysis and related methods to tackle a wide gamut of problems which are briefly summarized here. For example, COVID-19 was the focus of two studies. In one of them, López-Hernandez et al. used untargeted MS-based metabolomics to investigate dysregulations in lipid pathways 2 years after recovery from the disease, adding important insights into our understanding of long COVID-19. In the other, the effects of SARS-Cov-2 on lung parenchyma were compared to those caused by other severe pulmonary infections through analysis of the ^1^H NMR profiles of tissue extracts, corroborating that distinct metabolic signatures associated with energy metabolism and inflammatory pathways differentiate COVID-19 from other respiratory infections (Hurtado et al.). Through a translational study supported largely by untargeted NMR-based metabolomic analysis and targeted GC-EI-MS data, a multinational team led by Argentine researchers reported the identification of nicotinamide as a potential biomarker for Alzheimer’s disease (Dalmasso et al.). The application of untargeted metabolomics and lipidomics based on LC-QTOF-MS and GC-QTOF-MS found dysregulation of glycerolipid and sphingolipid metabolic pathways in the plasma of acute leukemia patients (Arévalo et al.). These differences are independent of lifestyle, race, or geographic location, providing valuable clues for the development of global therapies. NMR-based plasma metabolomics of individuals with differential responses to HIV-1 exposure and/or infection revealed that different pathways are affected in each group relative to controls (Gómez-Archila et al.). In particular, the study was the first to identify that HIV-1-exposed but seronegative (HESN) individuals have a specific metabolic fingerprint with significant alterations in LDL, glucose, lactate, and phosphocholine levels. Based on a systematic review of 26 independent metabolomics studies on systemic sclerosis, Morales-Gonzáles et al. identified 151 metabolites associated with the condition. Species linked to amino acid, lipid, and TCA cycle metabolic pathways are the most dysregulated. These confirm the impact of autoimmune inflammation, vascular damage, fibrosis, and gut dysbiosis in the progression of this disease, and also represent potential biomarkers for its early diagnosis and prognosis. The gut microbiome in a Colombian cohort of pregnant and lactating women was investigated by untargeted metabolomics based on LC-QTOF-MS coupled to molecular networking (Londoño-Osorio et al.). The report helps to identify metabolites with potential use in nutritional and physiological state assessments as well as personalized health and nutrition strategies. Using MS-based multiplatform metabolomics, Pardo-Rodriguez et al. investigated alterations in the metabolism of *Trypanosoma cruzi* after treatment with extracts of the Andean shrub *Clethra fimbriata*. More than 150 altered metabolites were identified in the treated parasites, with those related to energy metabolism pathways being the most affected. In addition, the authors found that triterpenes originating on the plant contributed to the disruption of essential processes in the parasite. Based on a study involving MS/MS data of beauvericins, depsipeptides present in *Fusarium* spp. Fungi, Selegato et al. demonstrated that the combination of feature-based molecular networking and MassQL is an effective strategy for accelerating the decoding of mass fragmentation pathways and identifying molecules with comparable fragmentation patterns. In combination with molecular networking analysis, MS-based untargeted metabolomics was applied to investigate the influence of altitudinal variations in the chemical composition of different bamboo species (Chivita et al.). The study uncovered 89 differential metabolites between the altitudinal ranges investigated, with an increase in the profile of flavonoids observed at high altitude and a boost in the levels of cinnamic acid derivatives registered at low altitude. Finally, Arrieta-Echeverri et al. carried out a characterization of the microbial populations and chemical space composition of a water kefir fermentation using culture-dependent methods, compositional metagenomics, and untargeted metabolomics based on LC-QTOF-MS. The work provides specific knowledge that could be easily applied to the rational development of novel probiotic and postbiotic ingredients for functional nutrition.

To conclude, it is worth mentioning that several of the manuscripts published in this Research Topic are the product of synergistic collaborations between Latin American research groups, and that those collaborations developed, in great part, from events fostered by LAMPS. We are already organizing the upcoming V LAMPS Meeting, which will include several workshops and will have the participation of invited experts from around the World. We are certain that this meeting, which will be held in Montevideo, Uruguay, from October 30th to 1 November 2024 (https://sites.google.com/unesp.br/v-lamps-2024/home), will confirm that the field of metabolomics, as well as its ancillary methodologies, continues to expand and mature in the region.

